# Idiopathic Mandibular Condyle Resorption

**DOI:** 10.7759/cureus.11365

**Published:** 2020-11-06

**Authors:** Christopher C Zarour, Ciji Robinson, Arooj Mian, Mohammed Al-Hameed, Michael Vempala

**Affiliations:** 1 Radiology, St. Joseph Mercy Hospital, Pontiac, USA; 2 Radiology, Ross University School of Medicine, Bridgetown, BRB; 3 Family Medicine, Windsor University School of Medicine, Basseterre, KNA; 4 Radiology (Diagnostic Radiology), St. Joseph Mercy Hospital, Pontiac, USA

**Keywords:** jaw pain, atrophic mandible, condyle, idiopathic, micrognathia, mandibular hypoplasia, temporomandibular joint (tmj) disorders

## Abstract

Idiopathic mandibular condylar resorption is a rare condition in which the mandibular condyle of the temporomandibular joint (TMJ) becomes resorbed and thus reduces in size and volume. This leads to TMJ dysfunction that commonly requires surgical correction; however, more conservative interventions can also be utilized. We present a case of idiopathic mandibular condyle resorption in a 17-year-old female presenting with TMJ pain and clicking with mastication. A definitive diagnosis of this condition ultimately requires imaging studies, a reliable option being magnetic resonance imaging (MRI), which will reveal erosion of the mandibular condylar process (often bilaterally) with diminished mass and volume leading to the known sequelae of symptoms.

## Introduction

Mandibular condylar resorption is a rare condition in which the condylar process of the mandible is progressively resorbed and thus reduces in size over a period of time [1]. This phenomenon can be categorized as either primary or secondary condylar resorption [2]. Some well-recognized secondary causes of mandibular condyle resorption include preexisting temporomandibular joint (TMJ) dysfunction, steroid use, trauma, prior surgery, and certain autoimmune diseases, such as rheumatoid arthritis, scleroderma, and systemic lupus erythematosus [2-3]. Primary mandibular condylar resorption can also be termed ‘idiopathic’ as the etiology remains unknown [3]. The mandibular condyle serves as the articulation point with the temporal bone which together comprises the TMJ. Their articular surfaces are separated by an articular disc that serves to provide a smooth and cushioned surface in which the joint can effectively move.

If the condylar process undergoes erosion at the TMJ, the joint naturally retracts posteriorly, resulting in mandibular retrognathia, malocclusion of the teeth, and open bite deformities [3-4]. Patients will commonly report symptoms, such as jaw pain, headaches, and a clicking and/or popping noise upon TMJ manipulation [4]. Reduction in the expected range of motion of the jaw can also be seen [5-6]. Mandibular condylar resorption has been observed in both men and women; however, it highly favors the female population (in a 9:1 ratio), showing a predominance in females undergoing pubertal changes [5]. Given this demographic, it has commonly been referred to as ‘cheerleader’s syndrome' [5].

## Case presentation

A 17-year-old female presented to her primary care physician with recurrent jaw pain and clicking upon TMJ manipulation. She had no significant past medical history at the time of presentation and did not experience any prior trauma or any surgeries in the past. She denied any other joint pain or systemic symptoms.

**Figure 1:**
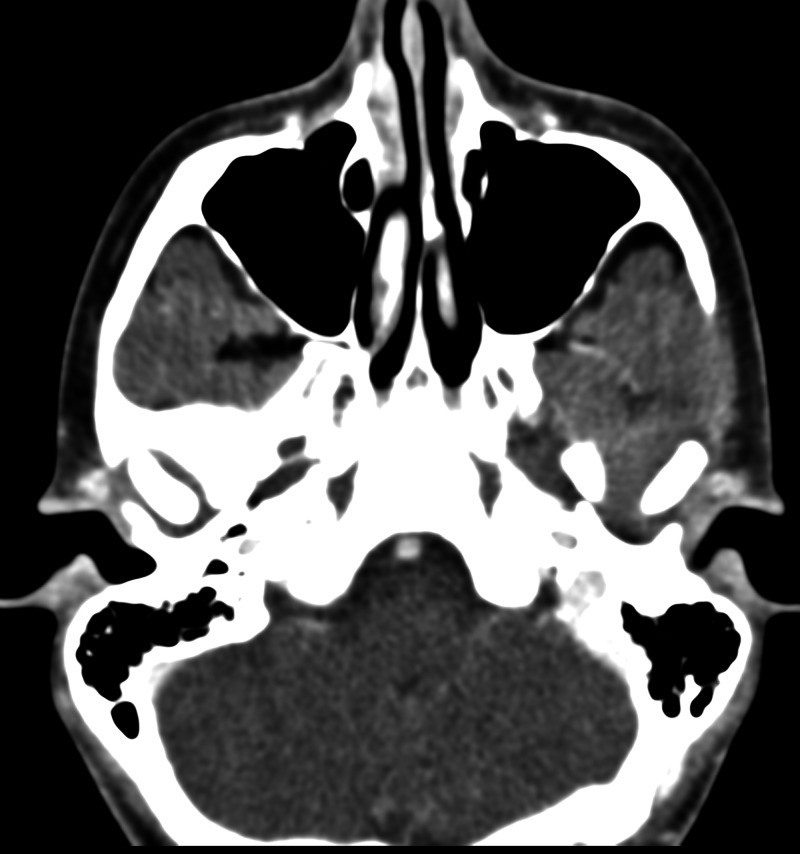
Axial non-contrast maxillofacial computed tomography (CT) (soft tissue window) demonstrates no soft tissue abnormality or mass

**Figure 2:**
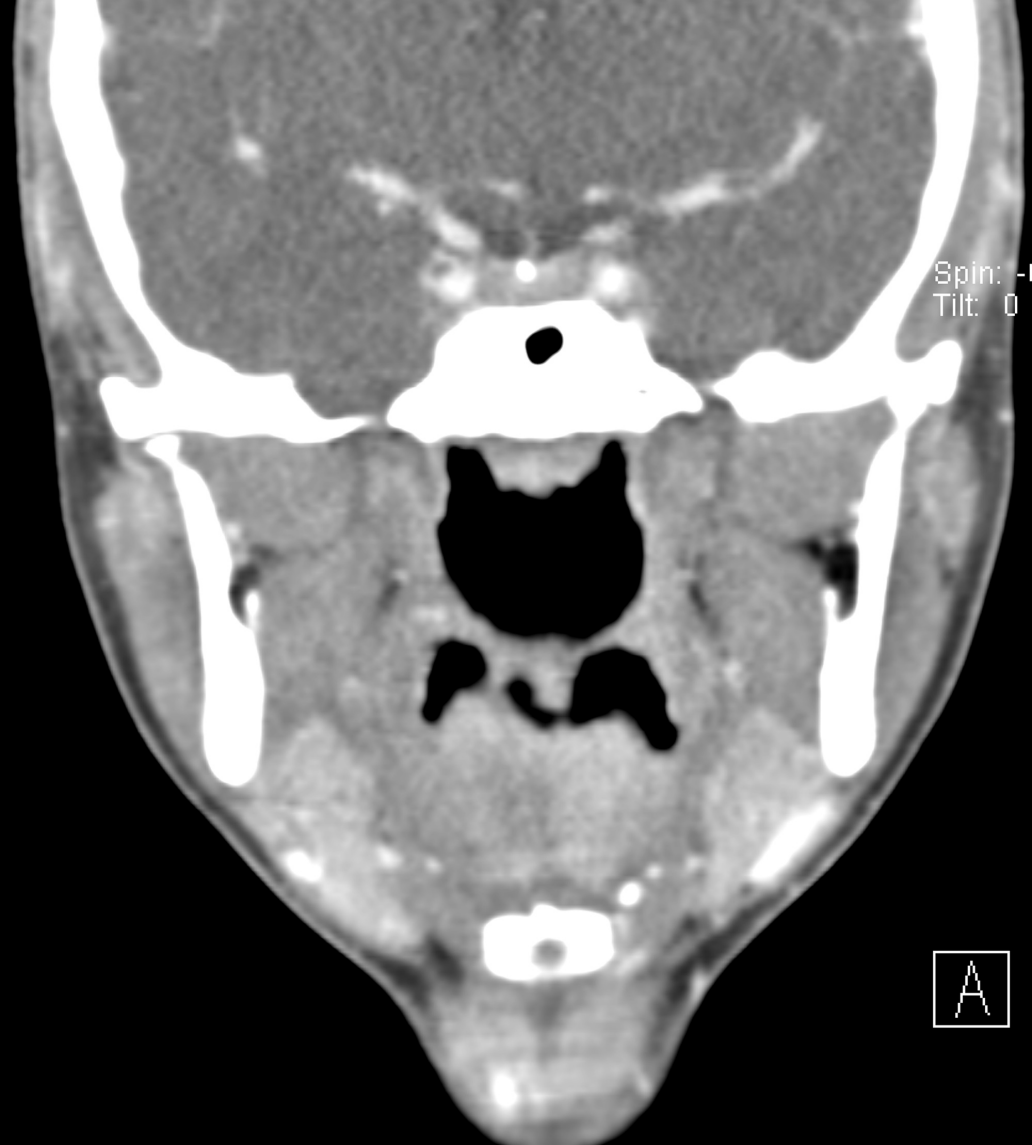
Coronal non-contrast maxillofacial computed tomography (CT) (soft tissue window) demonstrates no soft tissue abnormality or mass

**Figure 3:**
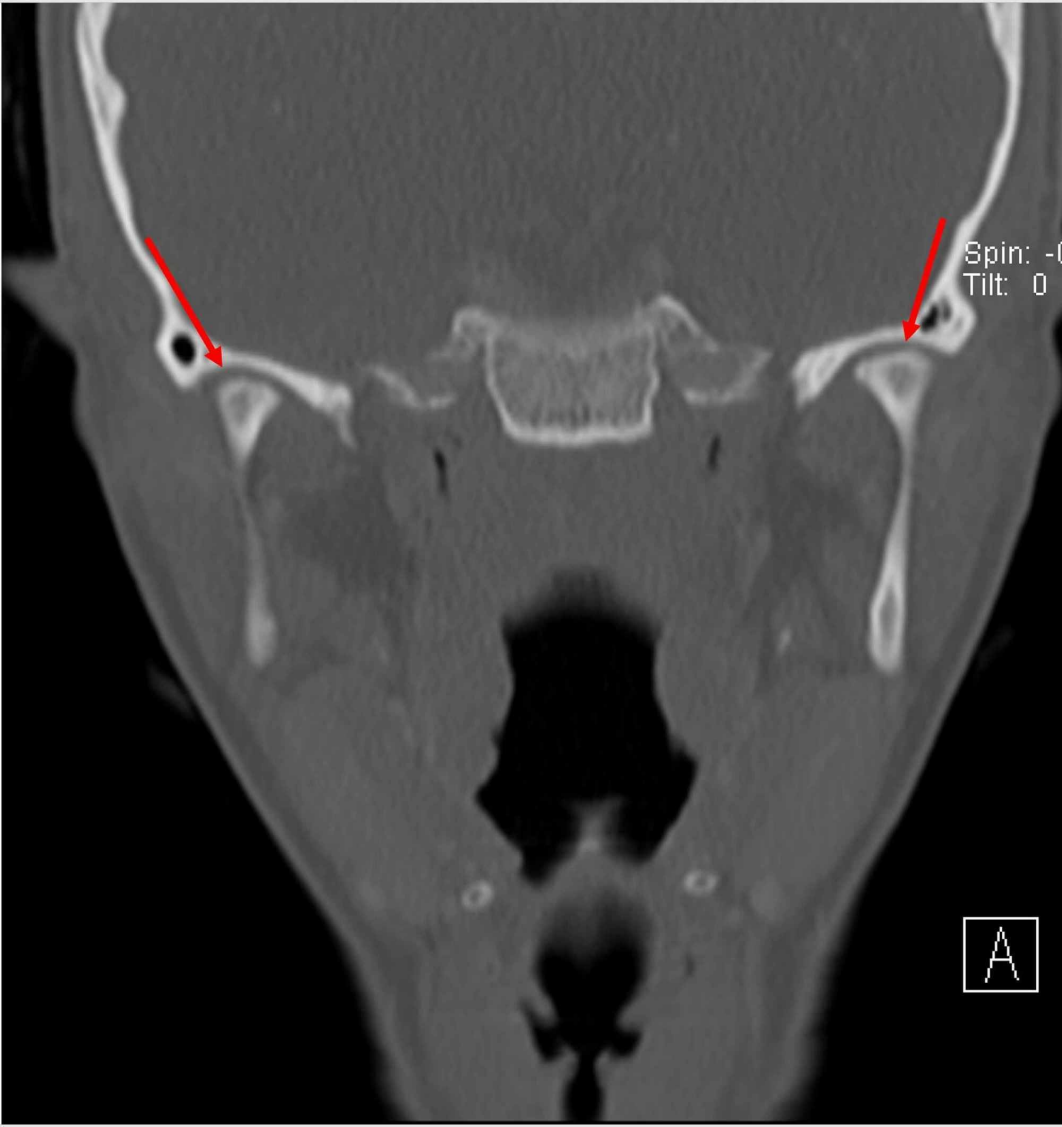
Coronal maxillofacial computed tomography (CT) (bone window) demonstrates resorption/flattening of the bilateral mandibular condyles and joint space narrowing of the bilateral temporomandibular joints

**Figure 4:**
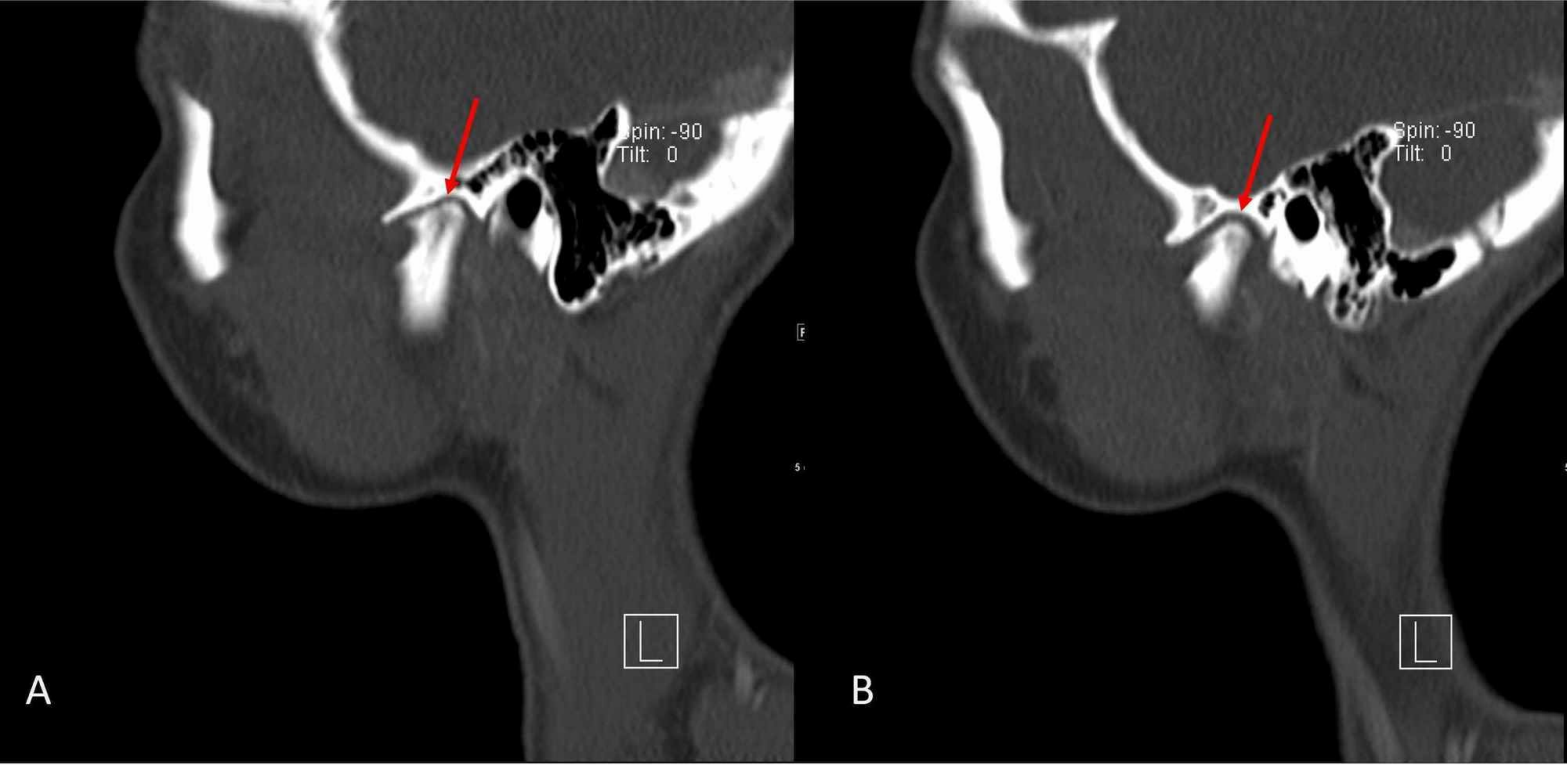
Sagittal non-contrast maxillofacial computed tomography (CT) (bone window) Left (A) and right (B) demonstrate resorption/flattening of the bilateral mandibular condyles with severe temporomandibular joint space narrowing.

Physical examination was unremarkable, except for induced pain and a clicking sound appreciated bilaterally upon TMJ movement. Maxillofacial computed tomography (CT) was ordered (Figures 1-4) which demonstrated bilateral mandibular condyle resorption/flattening and severe TMJ space narrowing.

## Discussion

Whether the bone loss that occurs in condylar resorption is due to hormonal influences, inflammation, or overwhelming compressional forces, there is a common resorption pathway involved (1). This pathway involves cytokines, e.g., tumor necrosis factor-alpha (TNF-α), interleukin-6 (IL-6), or receptor activator of nuclear factor kappa-B ligand (RANKL), that activate osteoblasts in the associated bone [1]. These osteoblasts then recruit osteoclasts which carry out the breakdown process of the bone [1]. The process can also be initiated by free radicals produced as a result of compressional forces within the TMJ from excessive joint loading [1].

Estrogen is known to play a role in bone and cartilage metabolism [1]. It has been shown that the TMJ and its articular disc both contain estrogen receptors, and elevated levels of these receptors have been identified in patients who have been diagnosed with mandibular condylar resorption [2-4]. This helps to understand the well-known link between condylar resorption and pubertal females. Increased amounts of estrogen receptors located within the TMJ and its articular disc contribute to the metabolism of bone, as well as surrounding cartilage and ligaments which help to stabilize the joint [2-4]. There also appears to be hyperplasia of the surrounding synovial tissue [4].

Given our patient’s lack of significant past medical history, trauma, or surgeries, we can assume she experienced primary (or idiopathic) mandibular condylar resorption. Her age and gender also help to further solidify this assumption as the hormonal changes seen in this demographic are thought to contribute to the idiopathic category of this condition. Idiopathic condylar resorption is ultimately a diagnosis of exclusion once other potential (secondary) causes have been ruled out [5].

Imaging studies are imperative in arriving at a definitive diagnosis of condylar resorption as the remainder of the diagnostic criteria are based on clinical findings and patient history. Magnetic resonance imaging (MRI) is a reliable imaging modality to identify changes related to condylar resorption, and findings will be remarkable for a notable decrease in condylar size and volume, which is often bilateral [4-5]. Anterior displacement of the TMJ articular disc, as well as significant thinning at the head of the condyle, can also be appreciated [6]. Increased synovial tissue can also be visualized within the joint space, which has been postulated to contribute to the inflammation and subsequent breakdown of surrounding ligaments that is seen [7].

There has been minimal evidentiary support for the use of pharmacologic agents in the treatment of condylar resorption [1]. Management of condylar resorption remains a controversial topic and can range from the use of splints to surgical correction [2]. Several surgical approaches have been utilized; however, a well-known and accepted method involves an approach in which the hyperplastic synovial tissue is first surgically removed, which then allows for the articular disc to be properly repositioned and stabilized in its correct location [2, 4]. The joint space is further stabilized with the use of artificial ligaments to replace the ligaments that have been degenerated surrounding the joint space [4]. Surgical repositioning of the TMJ itself, as well as any associated occlusal deformities (orthognathic surgery), is then performed [8].

## Conclusions

Idiopathic mandibular condylar resorption is a rare condition affecting predominantly females within the pubertal age range. Hormonal changes are thought to play a role in the pathogenesis of idiopathic condylar resorption, explaining the most commonly affected demographic identified in clinical practice. Although surgical correction is the mainstay of treatment, a deeper understanding is required before an etiologic-based treatment can be established.
